# Enhanced tumor growth inhibition by mesenchymal stem cells derived from iPSCs with targeted integration of interleukin24 into rDNA loci

**DOI:** 10.18632/oncotarget.16584

**Published:** 2017-03-28

**Authors:** Bo Liu, Fei Chen, Yong Wu, Xiaolin Wang, Mai Feng, Zhuo Li, Miaojin Zhou, Yanchi Wang, Lingqian Wu, Xionghao Liu, Desheng Liang

**Affiliations:** ^1^ The State Key Laboratory of Medical Genetics and School of Life Sciences, Central South University, Changsha, China

**Keywords:** rDNA locus, gene targeting, MSCs derived from iPSCs, IL24, anti-tumor

## Abstract

Induced pluripotent stem cells (iPSCs) are a promising source of mesenchymal stem cells (MSCs) for clinical applications. In this study, we transformed human iPSCs using a non-viral vector carrying the *IL24* transgene pHrn-*IL24*. PCR and southern blotting confirmed IL24 integration into the rDNA loci in four of 68 iPSC clones. We then differentiated a high expressing IL24-iPSC clone into MSCs (IL24-iMSCs) that showed higher expression of IL24 in culture supernatants and in cell lysates than control iMSCs. IL24-iMSCs efficiently differentiated into osteoblasts, chondrocytes and adipocytes. Functionally, IL24-iMSCs induced *in vitro* apoptosis in B16-F10 melanoma cells more efficiently than control iMSCs when co-cultured in Transwell assays. *In vivo* tumor xenograft studies in mice demonstrated that IL24-iMSCs inhibited melanoma growth more than control iMSCs did. Immunofluorescence and histochemical analysis showed larger necrotic areas and cell nuclear aggregation in tumors with IL24-iMSCs than control iMSCs, indicating that IL24-iMSCs inhibited tumor growth by inducing apoptosis. These findings demonstrate efficient transformation of iPSCs through gene targeting with non-viral vectors into a rDNA locus. The ability of these genetically modified MSCs to inhibit *in vivo* melanoma growth is suggestive of the clinical potential of autologous cell therapy in cancer.

## INTRODUCTION

Human mesenchymal stem cells (MSCs) are adherent adult stem cells with an ability to differentiate into various mesenchymal cell types like osteoblasts, adipocytes, and chondrocytes [1]. MSCs are also involved in immunosuppression and migrate to sites of inflammation and tumors [2–5]. Many studies have shown that MSCs can be used as cellular vehicles to carry anti-tumor agents to tumor sites and inhibit tumor growth in animal models [6–10]. However, loss of differentiation potential and decline of proliferation during *ex vivo* expansion has limited their clinical application [11, 12]. Recently, human induced pluripotent stem cells (iPSCs) have become promising alternate source of MSCs that can be used for autologous cellular therapy in cancer [13, 14].

While most studies have used viral vectors to modify MSCs, it is preferable to use non-viral methods to engineer therapeutic cells for safety considerations. However, their lack of integration into host genome has prevented long term expression that is necessary for clinical applications. However, the ribosomal DNA (rDNA) locus that consists of nearly 400 copies of the 45S pre-RNA (*rRNA*) gene clustered on the short arms of all five acrocentric chromosomes 13, 14, 15, 21, and 22 with high transcriptional activity has been explores as a candidate site for transgene integration and long term expression [15]. Previously we successfully targeted a non-viral vector into the rDNA locus of different human cell types including hepatocyte cell lines, MSCs and embryonic stem cells (ESCs) and demonstrated efficient expression of transgenes [16–18].

Interleukin 24 (*IL24*), also called melanoma differentiation associated gene 7 (*MDA7*), is a unique cytokine and tumor suppressor gene that belongs to the *IL10* cytokine family [19]. Human *IL24* is a single copy gene that is located on chromosome 1 (q32.2-q41) and encodes a protein of 206 amino acids [20]. It has demonstrated significant anti-tumor activity in pre-clinical animal models [21–28]. Overexpression of *IL24* selectively inhibits the growth of melanoma cells and induces apoptosis [29, 30].

In the present study, we transformed human iPSCs by targeting a non-viral vector (pHrn) containing *IL24* into the rDNA locus using transcription activator-like effector nickase (TALENickase). Further, we differentiated the transformed iPSCs into iMSCs and characterized their anti-melanoma properties, both *in vitro* and *in vivo*.

## RESULTS

### Construction of pHrn-*IL24*

**Figure 1:**
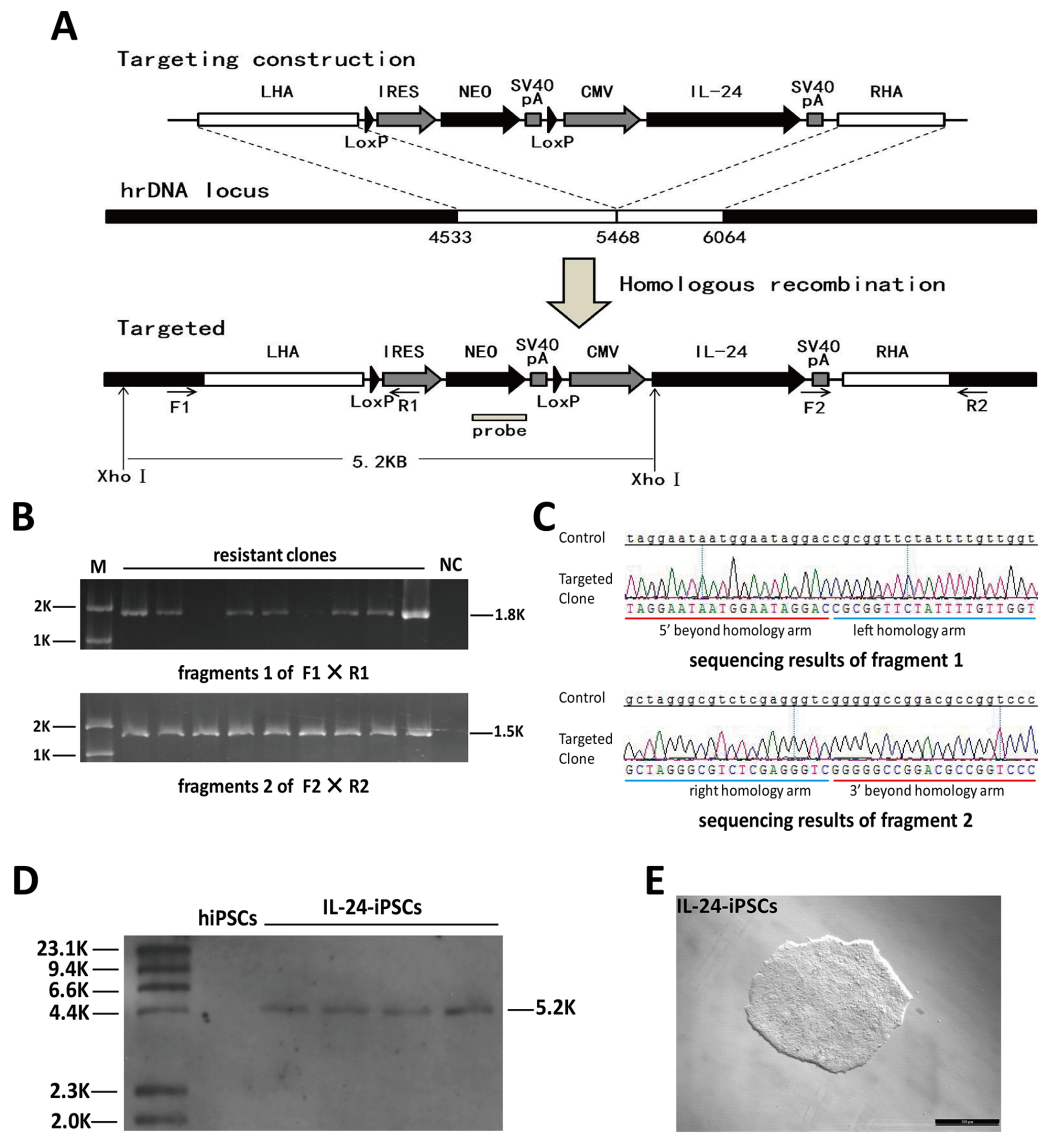
Analysis of site-specific integration of pHrn-*IL24* at the rDNA locus of iPSCs **(A)** Schematic representation of the gene targeting vector, the rDNA unit and targeted allele after homologous recombination is shown. The left (GenBank U13369: 4533–5467) and right (GenBank U13369: 5468–6064) homologous arms are shown by white boxes. The neomycin cassette consists of an IRES element from the encephalomyocarditis virus, the coding region of the neomycin gene (NEO), and SV40 polyA signal (SpA). Neo lacks a promoter. Gene expression is activated by the promoter of the endogenous rRNA genes after homologous recombination. The neomycin open reading frame (NEO ORF) was knocked-in into the rDNA unit resulting in an additional XhoI site that results in 5.2 kb fragment after digestion with XhoI. The *IL24* gene is driven by a CMV promoter. Primers F1/R1 bind to the 18S RNA coding sequence beyond the homologous sequence and the IRES element; primers F2/R2 bind to the SV40 polyA signal and the 5.8S RNA coding sequence beyond the homologous sequence, respectively. **(B)** PCR screening for the G418 resistant clones were screened by PCR with F1/R1 and F2/R2 pairs that would generate 1.8kb and 1.5kb PCR products, respectively, if the cells underwent homologous recombination. **(C)** DNA sequencing of the two PCR products is shown. Both fragments were consistent with the expected theoretical sequences. **(D)** The positive clones (based on PCR) were confirmed by Southern blot analysis of the XhoI digested genomic DNA. Southern blot analysis showed a 5.2 kb band for transformed iPSCs clones with the designed probe. **(E)** Morphology of a representative IL24-iPSCs clone. Abbreviations: M, markers; NC, negative control.

We constructed an optimized rDNA targeting plasmid, pHrn-*IL24* (Figure 1A), which contained a promoter-less neomycin resistance cassette and a cytomegalovirus (CMV) enhancer driven human *IL24* ORF cassette for integration into the 45S pre-RNA gene [16]. The two cassettes were flanked by a short 5′ left homologous arm (935 bp) and a short 3′ right homologous arm (596 bp). The first cassette contained an encephalomyocarditis virus internal ribosomal entry site (EMCV-IRES) that enabled gene expression under the control of an upstream endogenous RNA polymerase I promoter. LoxP sequences were inserted on either sides of the first cassette to enable insertion via homologous recombination. The plasmid was verified by DNA sequencing.

### Targeting of *IL24* at the rDNA locus in iPSCs

We previously designed TALENickase by introducing mutations into the FokI domain sequence that generates DNA single-strand breaks (SSB) [31]. TALENickase improved site-specific integration efficiency of pHrneo at the rDNA locus and demonstrated comparatively lower cytotoxicity and off-target mutagenesis compared to conventional double strand breaks (DSB) inducing TALENs. Therefore, pHrn-*IL24* and TALENickases were co-nucleofected into the iPSCs. Further, to improve single cell survival, we added 10μM Y-27632 (rho-associated kinase (ROCK) inhibitor) 2h before nucleofection into the medium and maintained it further for 24h after nucleofection. Then, after 6 days, we conducted two independent selection experiments with 50μg/ml G418 and obtained sixty eight G418-resistant clones. Finally, analysis of 31 G418-resistant clones PCR and southern blot demonstrated that four transformed iPSC clones had the target transgene (Figure 1B–1E).

### Expression of exogenous *IL24* in iPSCs

**Figure 2:**
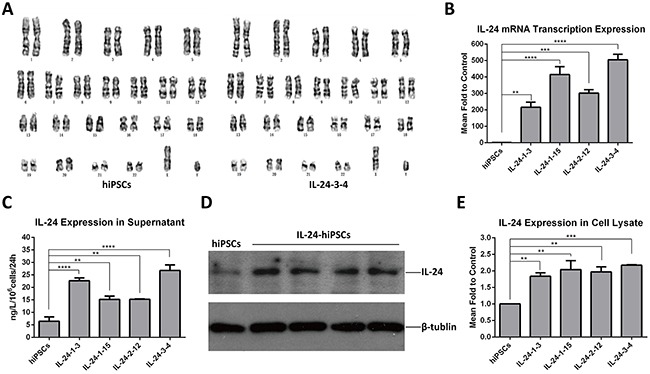
Analysis of IL24 expression in tranformed iPSCs **(A)** The transformed iPSC clones showed a normal karyotype (46, XY) (right) consistent with the normal iPSCs (left); n=5 for each group. **(B)** Detection of *IL24* mRNA transcription levels in control hiPSC and positively transformed hiPSC lines (IL-24-1-3; IL-24-1-15; IL-24-2-12; IL-24-3-4) by qRT-PCR. The IL24 levels in targeted iPSCs were higher than normal iPSCs. (Data are mean ± SEM, n = 3 for each group. ** p < 0.01, ***p < 0.001, **** p < 0.0001, One-way ANOVA.) **(C)** ELISA analysis of the supernatant of clones from control iPSC and positively transformed iPSC lines. The levels of secreted IL24 in different transformed iPSC clones was in the range of 15-26ng/10^6^ cells in 24h respectively. (Data are mean ± SEM, n = 4 for each group. ** p < 0.01, **** p < 0.0001, One-way ANOVA.) **(D** and **E)** Western blot analysis of cell lysates of clones from control hiPSC and transformed iPSC lines. The expression level of IL24 in the 4 transformed clones was about 2 fold higher than in iPSCs. (Data are mean ± SEM, n = 3 for each group. ** p < 0.01, ***p < 0.001, One-way ANOVA.).

The analysis of G-banded chromosomes in the four positive iPSC clones by genotyping showed that their karyotypes were normal and similar to normal iPSCs (Figure 2A). Then, we determined if the *IL24* transgene was expressed in the iPSCs by qRT-PCR with primers designed to amplify exons 6 and 7 of *IL24*. As expected, the transcription of *IL24* in the transformed iPSCs was significantly higher than in the control iPSCs (Figure 2B). Next, we determined the expression levels of IL24 protein in the supernatant and cell lysates by ELISA and Western blotting. Quantitative analysis showed that IL24 protein of targeted iPSCs was significantly increased compared to control iPSCs (Figure 2C to 2E). In the supernatant, IL24 in the transformed iPSCs was between 15–26ng /10^6^ cells compared to 6ng /10^6^cells in control iPSCs in 24h. In cell lysates, the expression of IL24 in transformed iPSCs was nearly 2 fold higher than the control iPSCs. These data demonstrated that the integrated *IL24* transgene expressed efficiently in the transformed iPSCs.

### Generation and characterization of MSCs derived from IL24-iPSCs and iPSCs

**Figure 3:**
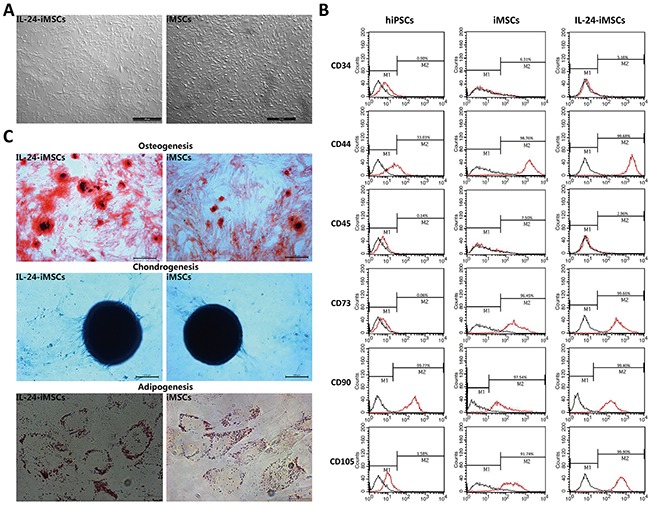
Generation and characterization of iMSCs **(A)** Morphology of the MSCs derived from IL24-iPSCs and control iPSCs. **(B)** Flow cytometric analysis of surface markers in human MSCs, iPSCs, iPSCs derived MSCs and IL24-iPSCs-derived MSCs. Black curves represent isotype controls and red curves represent the specific antibodies. (n=3 for each group). **(C)** Analysis of adipogenic, osteogenic, and chondrogenic differential potential of IL24-iMSCs and iMSCs. The osteogenic cultures were stained with alizarin red S (Cyagen Biosciences, China) to detect calcium deposition. For chondrogenic induction, the pellet sections were stained with alcian blue dye (Cyagen Biosciences, China) to detect proteoglycans. The adipogenic cultures were stained with oil red O (Cyagen Biosciences, China) to measure the accumulation of intracellular lipids. (n=3 for each group).

Then, we differentiated the IL24-iPSC line (IL24-3-4) with high IL24 expression and a control iPSC line into IL24-iMSCs and control iMSCs, respectively. During differentiation, IL24-iPSCs and control iPSCs were dissociated and treated with MSC differentiation medium containing epidermal growth factor (EGF) and platelet derived growth factor BB (PDGF-BB), both of which stimulate differentiation of iPSCs into MSCs. After 2 weeks of differentiation, confluent cells were passaged and cultured on gelatinized plates with MSC culture media and allowed to proliferate. We observed that after 4 passages, the induced iPSCs uniformly displayed fibroblast-like morphology that was similar to MSCs (Figure 3A) and different from iPSCs. Further, analysis of cell surface markers of IL24-iMSCs and iMSCs by flow cytometry showed that they were CD44^+^CD73^+^CD90^+^CD105^+^CD34^−^ CD45^−^ and similar to human bone marrow MSCs (Figure 3B). We then tested the differentiation potential of IL24-iMSCs and iMSCs and found that nearly 80-90% cells were positive for osteogenic, chondrogenic and adipocytic differentiation based on histochemical staining (Figure 3C). These results demonstrated that IL24-iPSCs and iPSCs differentiated efficiently into MSCs.

### IL24-iMSCs induce melanoma cell apoptosis *in vitro*

**Figure 4:**
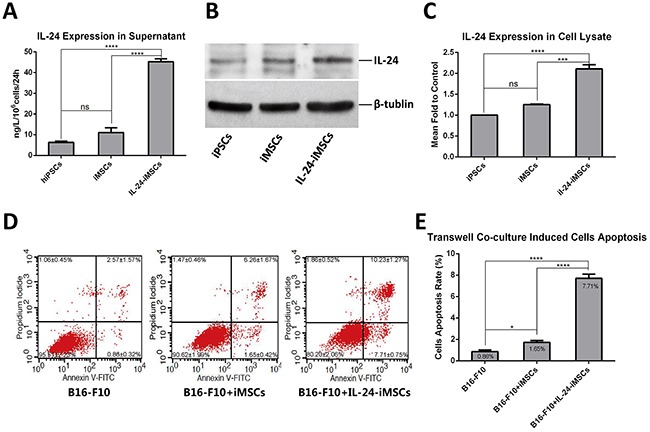
IL24-iMSCs induce *in vitro* melanoma cell apoptosis **(A)** ELISA analysis of IL24 in the supernatants from iMSCs and IL24-iMSCs is shown. IL24 levels in the supernatants of iPSCs, iMSCs and IL24-iMSCs were 6.33±0.97ng, 10.58±2.66ng and 45.18±2.53ng, respectively in 10^6^ cells in 24h. (Data are mean ± SEM; n = 4 for each group; ns, p > 0.05; ****, p < 0.0001 by Oneway ANOVA). **(B** and **C)** Western blot analysis of cell lysates from control iMSCs and IL24-iMSCs. The expression levels of IL24 in IL24-iMSCs were about 2 fold higher than in control iMSCs.(Data are mean ± SEM; n = 3 for each group; ns, p>0.05, ***, p<0.001, ****, p<0.0001, One way ANOVA). **(D** and **E)** Flow cytometric analysis of cell apoptosis assay. B16-F10 cells co-cultured with IL24-iMSCs showed increased apoptosis compared to co-culturing with control iMSCs or control B16-F10 cells *in vitro*. (Data are mean ± SEM; n = 3 for each group; * p < 0.05, **p < 0.01, ***p < 0.001, ****p < 0.0001, One way ANOVA).

IL24 expression was quantitated by ELISA and western blot analysis in IL24-iMSCs. IL24 levels were 4.2 fold higher in the supernatant of IL24-iMSCs than control iMSCs and 7 fold higher than in the control iPSCs (Figure 4A–4C). Comparatively, IL24 expression was 2 fold higher in the IL24-iMSC cell lysates compared to control iMSCs (Figure 4A–4C). However, no significant difference was observed in IL24 expression between control iPSCs and iMSCs suggesting that differentiation of iPSCs into MSCs did not increase endogenous levels of IL24.

Then, we determined if the IL24-iMSCs efficiently induced *in vitro* melanoma cell apoptosis by co-culturing IL24-iMSCs and B16-F10, a mouse melanoma cell line in a transwell system. After 7 days of co-culturing, cells were stained with AnnexinV-FITC/PI and B16-F10 cell apoptosis was analyzed by flow cytometry. We observed that the apoptosis rate of B16-F10 cells co-cultured with IL24-iMSCs was nearly 4.6 fold higher than B16-F10 cells co-cultured with control iMSCs and 8.9 fold higher than B16-F10 cells cultured alone (Figure 4D–4E). These data indicated that both IL24-iMSCs and iMSCs induced *in vitro* apoptosis in melanoma cells and IL24-iMSCs were more effective than the control iMSCs.

### IL24-iMSCs inhibit *in vivo* melanoma growth

**Figure 5:**
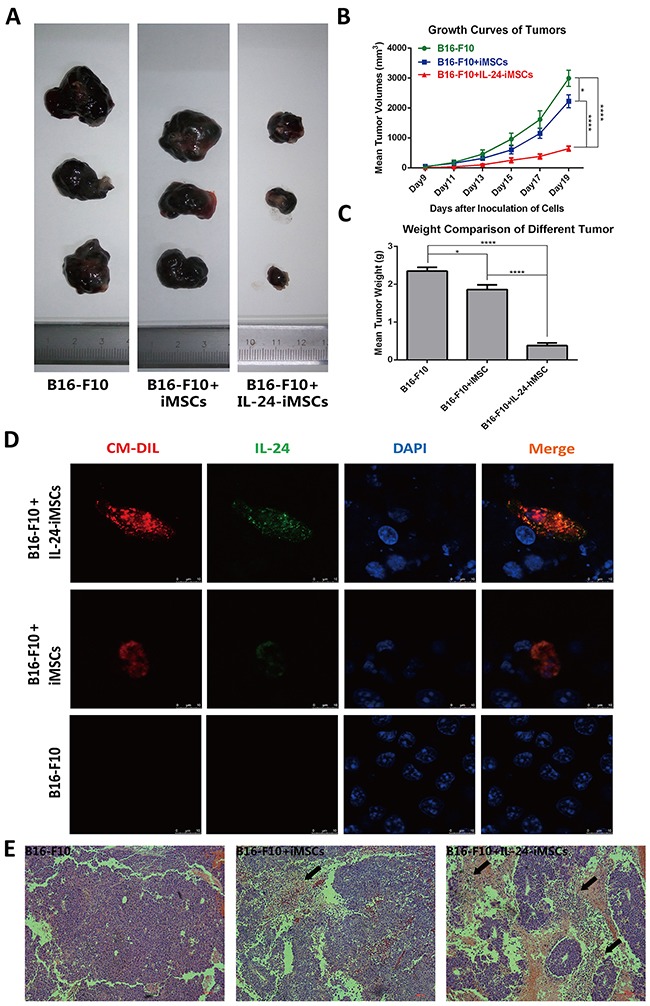
IL24-iMSCs *in vivo* suppressed melanoma growth **(A)** Representative images of isolated tumors from mice injected with B16-F10 cells only, mixture of B16-F10 cells and iMSCs and mixture of B16-F10 cells and IL24-iMSCs at the time of sacrifice.(n≥4 for each group) **(B)** Time course of tumor volume changes in mice injected with or without IL24-iMSCs at 19 days. Tumor sizes of mixture of B16-F10 cells and IL24-iMSCs group were significantly reduced. (Data are mean ± SEM; n≥4 for each group; * p < 0.05, ****p < 0.0001, One way ANOVA). **(C)** Measurement of tumor weights from C57BL/6 mice injected with or without IL24-iMSCs at the time of sacrifice. Tumor weights of mixture of B16-F10 cells and IL24-iMSCs group were significantly reduced. (Data are mean ± SEM; n≥4 for each group; * p < 0.05, ****p < 0.0001, One way ANOVA). **(D)** Immunofluorescence analysis with human specific IL24 antibody of tumor tissue sections derived from mice injected with B16-F10 cells in combination with either IL24-iMSCs or control iMSCs, or only B16-F10 cells. Melanoma sections examined for CM-Dil fluorescence (red), human IL24 protein fluorescence (green) and the cell population by DAPI staining (blue). **(E)** H&E stained histological sections of tumors show more diffuse necrotic areas (arrow) in melanoma injected with IL24-iMSCs compared with control iMSCs group. (n = 3 for each group).

For *in vivo* studies, IL24-iMSCs and iMSCs were labeled with the fluorescent dye CM-Dil with > 90% efficiency. Previous studies have reported that CM-Dil does not alter viability, proliferation or differentiation of cells [32, 33]. To determine the *in vivo* effect of IL24-iMSCs on tumorigenesis, C57BL/6 mice were injected with mouse melanoma cells (B16-F10 cell line) and an equal number of CM-Dil labeled IL24-iMSCs or control iMSCs (n=4 each). Control mice were transplanted with CM-Dil labeled IL24-iMSCs only (n = 4) or CM-Dil labeled iMSCs only (n = 4) or B16-F10 cell line only (n = 6). Mice transplanted with either IL24-iMSCs or control iMSCs alone did not form any tumors. Mice transplanted with the B16-F10 cell line only formed tumors by day 7 with an average tumor volume of 2921.50 mm^3^ on day 19. Mice injected with both B16-F10 cells and iMSCs developed detectable tumors on day 7 with an average tumor volume of 2223.51 mm^3^ on day 19. In contrast, mice injected with both B16-F10 cells and IL24-iMSCs developed detectable tumors on day 9 with an average tumor volume of 643.64 mm^3^ on day 19, thereby demonstrating the inhibitory effects of IL24-iMSCs on tumor development (Figure 5A). Tumor weights were recorded immediately upon harvesting from mice on day 19. The volume of tumor in the control mice injected with B16-F10 cells was 3.6 fold larger than mice injected with both B16-F10 cells and IL24-iMSCs and 1.4 fold larger than mice injected with both B16-F10 cells and iMSCs (Figure 5B). Meanwhile, the weight of tumor in the control mice injected with B16-F10 cells was 6.3 fold larger than mice injected with both B16-F10 cells and IL24-iMSCs and 1.3 fold larger than mice injected with both B16-F10 cells and iMSCs (Figure 5C).

Immunofluorescence analysis of the tumors revealed that CM-Dil-positive IL24-iMSCs expressed human IL24 in the mouse melanomas (Figure 5D). *In situ* pathological examination of H&E stained tumor tissues from the mice injected with B16-F10 cells and either IL24-iMSCs or iMSCs showed necrotic areas and aggregation of cell nuclei in the tumor area (Figure 5E). Comparatively, the necrotic areas in the tumors from the IL24-iMSC group were larger than the iMSC group suggesting the apoptotic effects of transgenic IL24.

## DISCUSSION

Nearly two-thirds of the clinical trials in gene therapy are aimed at the treatment of various types of cancers and in viral vectors are used in most cases [34]. However, issues regarding safety concerns and loss or silencing of the inserted transgenes are encountered when viral vectors are used. In previous studies, we developed a non-viral gene targeting system that efficiently inserted into the rDNA locus of multiple cell types, including human ESCs and MSCs [16–18]. Engineered nucleases such as TALENs profoundly increase gene targeting frequency via double-strand break (DSB) [35]. However, since multiple DSBs generated by TALENs in multiple rDNA loci are likely to be fatal, we previously generated TALENickases which led to less off-target mutagenesis and cytotoxicity by creating SSB, it was more effective than TALENs in improving gene targeting at the rDNA locus [31]. In the present study, we efficiently targeted *IL24* into the rDNA locus of human iPSCs using the rDNA targeting vector and TALENickases, with a relative targeting efficiency of nearly 13% in two independent experiments.

Currently, there exist concerns regarding if MSCs suppress or promote tumor growth. Although some studies have demonstrated that MSCs migrate to tumor sites and promote tumor growth, the underlying mechanisms remain obscure [36, 37]. However, owing to their ability to migrate to tumor locations, MSCs represent promising tools to deliver therapeutic agents to cancer sites [8, 38–44]. Many studies have demonstrated that genetically engineered MSCs inhibit tumor growth in lung cancer, melanoma, glioma, liver cancer and breast cancer [41, 42, 45, 46]. In a previous study we targeted neomycin gene into the rDNA locus of hBM-MSCs and generated stably transformed hBM-MSCs with G418 selection [17]. We also demonstrated proliferation of transformed hBM-MSCs upon culturing [17]. Therefore, a new strategy was necessary to rapidly obtain abundant transformed MSCs for clinical applications, whenever necessary.

The purpose of this study was to verify if MSCs derived from transformed iPSCs could serve as cellular vehicles to exert anti-tumor effects *in vivo*. Many studies demonstrated that IL24, a cytokine of the IL-10 family that was originally identified from metastatic human melanoma cells [20], induced cancer cell apoptosis [19, 43, 47–49]. There is increasing evidence that IL24 inhibits growth of many diverse human cancers including melanomas [29], breast cancer [44], pancreatic cancer [50], gliomas [51], hepatocellular cancers [52] and ovarian cancer [53] without adversely affecting normal cells or tissues. Therefore, we chose *IL24* as the therapeutic gene in this study.

Our results revealed that both transformed iPSCs (IL24-iPSCs) and subsequently derived IL24-iMSCs efficiently expressed exogenous IL24 while retaining their cellular characteristics. Furthermore, both IL24-iMSCs and iMSCs induced apoptosis in melanoma cells, thereby inhibiting melanoma growth *in vivo*. Our results are in accordance with other findings that have demonstrated anti-tumor properties of factors released from MSCs [42, 45]. More importantly, IL24-iMSCs suppressed tumor growth more effectively than control iMSCs demonstrating the anti-tumor effects of transgenic IL24. Therefore, we postulate that this non-viral gene targeting system in iPSCs and subsequent iMSCs represent great advantage in terms of safety and stability of IL24 expression and therefore need to be pursued further.

In summary, we targeted *IL24* into the rDNA locus of human iPSCs using a combination of non-viral vector and TALENickases. Our studies demonstrated therapeutic efficacy of MSCs derived from the transformed iPSCs (with *IL24* gene) in a mouse melanoma model. Thus, we provide the first proof of principle of generating transformed iPSCs by gene targeting with a non-viral vector and subsequent generation of genetically modified MSCs with enormous clinical potential for autologous cellular therapy in cancers.

## MATERIALS AND METHODS

### Cell culture

Human induced pluripotent stem cells (DYR0100) were purchased from ATCC and cultured in mTeSR1 medium (STEMCELL Technologies, Canada) at 37°C in a humidified chamber maintained at 5% CO_2_. DYR0100 were passaged every 5 days [54] before being plated onto a Matrigel (BD Biosciences, USA) coated dishes. The MSCs derived from iPSCs were cultured in MSC medium with DMEM/LG (HyClone, USA) supplemented with 10% FBS (Gibco, USA). PMEF-NL fibroblasts (Millipore) were cultured in DMEM/HG (HyClone, USA) supplemented with 5% FBS, 2mM L-glutamine (Invitrogen, USA) and 1% non-essential amino acids (Invitrogen, USA). The murine melanoma cells B16-F10 (from a C57BL/6 strain) were purchased from ATCC and cultured in DMEM/HG supplemented with 10% FBS.

### Construction of pHrn-*IL24* plasmid and transformation of iPSCs

In our previous study, we constructed the non-viral human ribosomal DNA (hrDNA) targeting vector, pHrneo [16]. In this study, we optimized pHrneo by shortening the size of its left and right homology arms to 953bp and 596bp, respectively and inserted a pair of loxP sequences on both sides of the neomycin expression cassette. Then, we PCR amplified the *IL24* open reading frame (GenBank No. BC009681 249–872) from pHr-CMG vector constructed by our group [55], and inserted into the NheI restriction site of the optimized targeting vector to generate pHrn-*IL24*.

For gene targeting, the iPSCs were incubated at 37°C with TrypLE Select (Invitrogen, USA) for 3 min before harvesting and counting. Then, 3×10^6^ iPSCs were resuspended in 100μL nucleofection buffer (Human Stem Cell Nucleofector Kit 2, Lonza, Switzerland) with 5μg linearized pHrn-*IL24* and 5μg TALENickases as previously described [31]. Nucleofection was completed by NucleofectorII (Lonza, Switzerland) with program B016. The transfected cells were maintained on mitomycin-C treated PMEF cells in DMEM/F12 medium supplemented with 20% knockout serum replacement (Gibco, USA), 2mM L-glutamine, 1% non-essential amino acids, 0.1mM β-mercaptoethanol (Gibco, USA) and 10ng/ml basic fibroblast growth factor (Invitrogen, USA). On the 6th day after transfection, selection was initiated by adding 50μg/ml G418 (Sigma-Aldrich, USA) and grown for 9 days in selection media. The G418 resistant clones were then picked mechanically and expanded on Matrigel in mTeSR1 medium.

### PCR and qRT-PCR

Genomic DNA was isolated from iPSCs by phenol/chloroform extraction method. PCR was performed using LA Taq DNA polymerase (TaKaRa, Japan) according to the manufacturer's recommendations. To identify the site-specific integration colonies, the following pairs of primers were used: F1:5′-CCTGAGAAACGGCTACCACATCC-3′/ R1:5′- CATCCAAGGAAGGCAGCAGGC-3′; F2:5′-GAGCA TTCAAACAGTTGGACG-3′/ R2:5′-GATCCACCGCT AAGAGTCGTAC-3′. The annealing Temperature of F1R1 and F2R2 are 59°C and 58°C. Total RNA was extracted using TRIzol reagent (Sigma-Aldrich, USA) and treated with DNaseI (Thermo Fisher Scientific, USA) to eliminate genomic and other DNA. The qRT-PCR for each 50ng RNA sample was performed using HiScript II One Step qRT-PCR SYBR Green Kit (Vazyme, China) on Bio-Rad CFX96 touch qPCR system (Bio-Rad, USA) according to the manufacturer's instructions. The RNA samples were subjected to 40 cycles of amplification after 30 min of reverse transcription at 50°C. The data analysis was performed using the Bio-Rad CFX Manager software (Bio-Rad, USA). The hypoxanthine phosphoribosyltransferase (HPRT) gene was amplified as an internal control. The *IL24* qRT-PCR primers are as follows: 5′-CAGGCGGTTTCTGCTATTC-3′/ 5′-GAATTTCTGCATCCAGGTCA-3′. The annealing Temperature of primers is 60°C.

### Southern blotting

5μg genomic DNA was digested with XhoI restriction enzyme (New England Biolabs, USA) overnight and then electrophoresed on a 0.8% agarose gel for 3h at 180V followed by transfer to a positively charged nylon membrane (Roche Diagnostics, Switzerland) by capillary siphon blotting overnight. DNA molecular weight marker III, digoxigenin (DIG) -labeled DNA (Roche Diagnostics, Switzerland) and lambda DNA Hind III (TaKaRa, Japan) were used as molecular weight markers. The blots were then blocked and then hybridized with DIG-dUTP labeled probes overnight at 42°C. This was followed by incubation with AP-conjugated DIGantibody (Roche Diagnostics, Switzerland) for 30 min. After thorough washing, the signals were detected using CDP-Star (Roche Diagnostics, Switzerland) as a substrate for chemiluminescence. Probes were generated by PCR DIG Probe Synthesis Kit (Roche Diagnostics, Switzerland) using the primer pairs, 5′-GCCGAGAAAGTATCCATCA-3′/ 5′-CAGAGTCCCGCTCAGAAG-3′.

### Karyotyping

Three-day-old iPSCs and IL24-iPSCs cell clumps were treated with 0.08μg/ml colcemid (Sigma-Aldrich, USA) for 2.5h. The cells were then trypsinized, centrifuged, and incubated in 0.075M KCl for 30 min at 37°C. After fixing with Carnoy's fixative (Methanol: Acetic Acid = 3:1, Sinopharm Chemical Reagent, China), metaphase chromosome spreads were prepared using air drying.

### ELISA

After culturing iPSCs in mTeSR1 medium for 3 days, 24h-old supernatants were collected from six-well plates. Total cells were trypsinized and counted. All supernatants were collected in triplicate. ELISA was performed using paired antibodies for ELISA-IL24 (dilution of 1:100, goat polyclonal, Catalog# AF1965, R&D Systems, USA) according to the manufacturer's instructions.

### Western blotting

Tatal lysate samples of iPSCs and MSCs were prepared by RIPA Lysis Buffer (Beyotime, China) and quantified by BCA Protein Quantification Kit (Beyotime, China). Each 20μg cell protein lysate sample was loaded for electrophoresis for 2h at 120V and electrotransferred for 1.5h at 290mA onto PVDF membranes (Millipore, USA). Membranes were incubated overnight with primary anti-human IL24 antibody (dilution of 1:1000, goat polyclonal, Catalog# AF1965, R&D Systems, USA) and anti-human β-Tubulin antibody (dilution of 1:5000, mouse monoclonal, Catalog# T8328, Sigma-Aldrich, USA) at 4°C. After thorough washing, the membranes were incubated with the horseradish peroxidase-conjugated anti-goat (dilution of 1:10000, Catalog# 805-035-180, Jackson ImmunoResearch) and anti-mouse secondary antibodies (dilution of 1:10000, Catalog# 115-035-146, Jackson ImmunoResearch) for 1h at room temperature and detected by autoradiography using the ECL system (Millipore, USA). Prestained molecular weight standards (Thermo Fisher Scientific, USA) were used to estimate the apparent molecular weight.

### Generation and characterization of MSCs derived from iPSCs

For differentiation of iPSCs into MSCs, we optimized an established protocol [14]. Briefly, a confluent 6cm dish of iPSCs was trypsinized and seeded at a density of 1×10^4^ cells/cm^2^ in a matrigel coated 6-well tissue culture plate in mTeSR1 medium. When the cells reached 60%–70% confluence, the culture medium was changed to MSC differentiation medium that was composed of knockout DMEM (Gibco, USA) supplemented with 10% knockout serum replacement, 1% non-essential amino acids, 2mM L-glutamine, 0.1mM β-mercaptoethanol, 20ng/ml bFGF, 20ng/ml EGF (Life Technologies, USA) and PDGF-BB (PeproTech, USA). After three passages, the differentiated cells were trypsinized and seeded in gelatinized 6-well tissue culture plates in MSC culture medium (DMEM/LG supplemented with 10% FBS and 2mM L-glutamine).

The surface antigen profiling of IL24-iMSCs and iMSCs was characterized by flow cytometry after incubated 30min with fluorescently tagged anti-human PE-CD34, PE-CD44, PE-CD45, PE-CD73, FITC-CD90, and PE-CD105 (dilution of 1:20, Catalog# 550761, 550989, 555483, 550257, 555595, 560839, BD Biosciences, USA) at 37°C according to the manufacturer's instructions. Differentiation of IL24-iMSCs and iMSCs was carried out using Osteogenesis, Chondrogenesis and Adipogenesis differentiation kits (Invitrogen, USA) according to the manufacturer's protocol.

### Apoptosis detection

To quantify apoptosis, 3×10^4^ B16-F10 cells were seeded in the upper transwell chamber of a 6well transwell plate (0.4μm PET MEM, Corning, USA) and 9×10^4^ IL24-iMSCs or control iMSCs were seeded in the lower transwell chamber. The plates were incubated at 37°C and 5% CO_2_. After 7 days of co-culturing in the same medium, B16-F10 cells were harvested and stained with AnnexinV-FITC/PI Apoptosis Detection Kit (Vazyme, China) according to the manufacturer's instructions followed by flow cytometry and analysis to estimate the percentage of apoptotic cells (AnnexinV-positive).

### Mouse xenograft assays and iMSC cell tracking in tumors

IL24-iMSCs and iMSCs were labeled with 5μM CM-Dil (Invitrogen, USA) according to the manufacturer's instructions. After labeling, cells were washed with PBS and cultured in MSC culture medium. The labeling efficiency was examined with a fluorescent microscope. Male C57BL/6 mice (4-6 weeks old) were obtained from the Center of Experimental Animals, Shanghai Institutes for Biological Sciences and kept in pathogen free conditions. All animal procedures were approved by the Ethics Committee for Animal Experimentation of Central South University. The mice were divided into five groups (n > 4, for each group). The test group was treated with a mixture of CM-Dil labeled IL24-iMSCs (5×10^5^) and B16-F10 cells (5×10^5^). In the control groups, CM-Dil labeled IL24-iMSCs (5×10^5^), CM-Dil labeled iMSCs (5×10^5^), B16-F10 cells (5×10^5^), CM-Dil labeled iMSCs (5×10^5^) mixed with B16-F10 cells (5×10^5^) were injected separately. For all groups, cells were resuspended in 0.1ml medium and subcutaneously injected into the groin region of anesthetized C57BL/6 mice. Tumor size was measured by vernier calipers every 2 days, and tumor volume was calculated with the formula [V = (W^2^ × L)/2] in cubic millimeters, where W and L are the shortest and longest diameter.

### Immunofluorescence and histological analysis

The mice were sacrificed on the 19^th^ day after xenografting by cervical dislocation under anesthesia with diethyl. Tumors were harvested, photographed, weighed, and fixed with 4% paraformaldehyde. Half of the tumors were used for frozen sections (10μm thickness). For immunofluorescence detection of IL24, sections were incubated with anti-human IL24 antibody (dilution of 1:100, goat polyclonal, Catalog# AF1965, R&D Systems, USA), followed by incubation with fluorescent AF488-anti-goat secondary antibody (dilution of 1:100, Catalog# 205-545-108, Jackson ImmunoResearch). CM-Dil and fluorescence staining was visualized and acquired by the confocal fluorescent microscopy. Paraffin sections (4μm thickness) were stained with hematoxylin and eosin (H&E) for histological examination.

### Statistical analysis

The Student's t-test and one-way ANOVA were used for data analysis of different experimental groups by GraphPad Software. Data were expressed as the mean ± standard error of the mean. P < 0.05 was considered statistically significant.
